# Supplementary material for: The Gut Microbiome, Seleno-Compounds, and Acute Myocardial Infarction
Source: J Clin Med. 2022 Mar 7;11(5):1462. doi: 10.3390/jcm11051462 (PMC8911090; doi:10.3390/jcm11051462)
Supplement: Supplementary file 1 [file jcm-11-01462-s001.zip › jcm-1525269-supplementary.pdf]

Journal of
Clinical Medicine

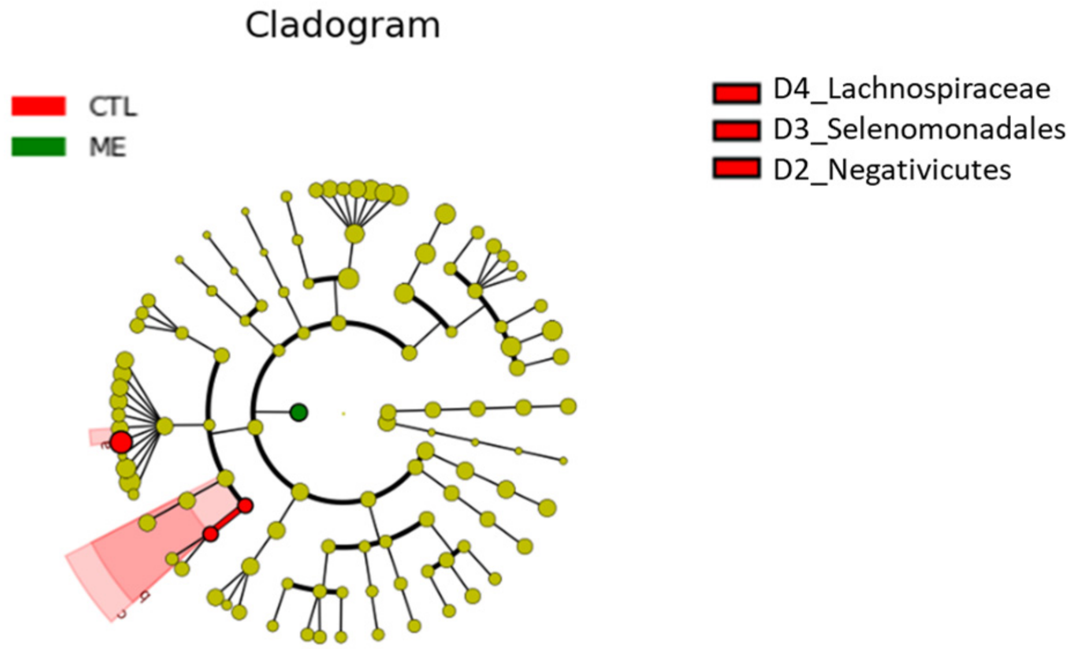

Figure S1. Cladogram showing different abundant taxa at Family level. Alphabets correspond to those in parentheses (CTL: control; ME: AMI).

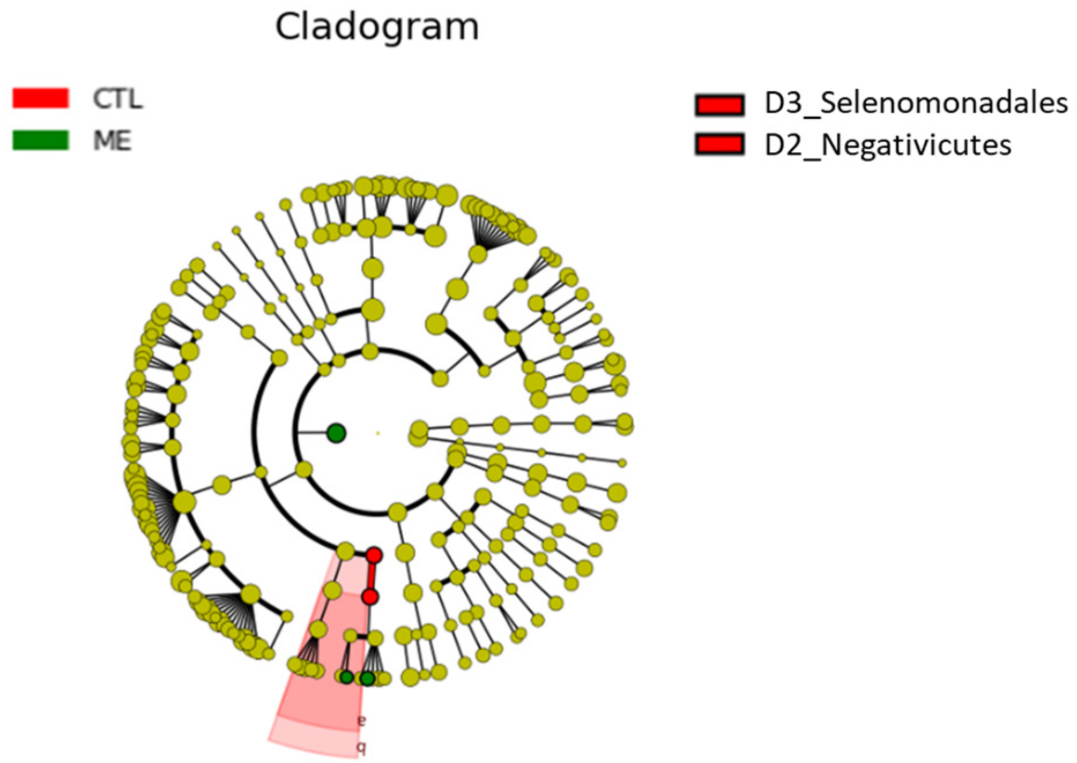

Figure S2. Cladogram showing different abundant taxa at Genus level. Alphabets correspond to those in parentheses (CTL: control; ME: AMI).

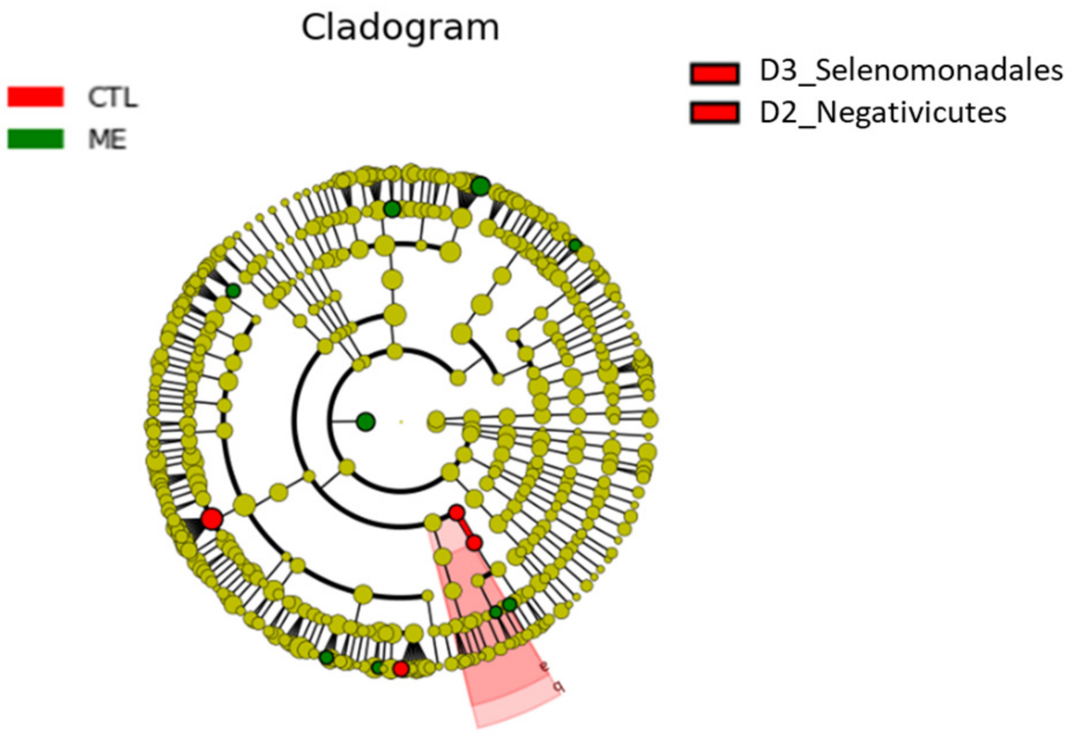

Figure S3. Cladogram showing different abundant taxa at Species level. Alphabets correspond to those in parentheses (CTL: control; ME: AMI).
